# Special Issue “IFN-Independent ISG Expression and Its Role in Antiviral Cell-Intrinsic Innate Immunity”

**DOI:** 10.3390/v11110981

**Published:** 2019-10-24

**Authors:** Emmanuel Thomas, Takeshi Saito

**Affiliations:** 1 Department of Microbiology and Immunology, University of Miami Miller School of Medicine, Miami, FL 33136, USA; 2 Department of Medicine, Division of Gastrointestinal and Liver Diseases, Molecular Microbiology & Immunology, and Pathology, Keck School of Medicine, University of Southern California, Los Angeles, CA 90033, USA

Over the last few decades, accumulating evidence has demonstrated that numerous Interferon (IFN) stimulate genes (ISGs) can be directly upregulated following virus infection independent of IFN signaling. Indeed, whereas typical ISGs are driven by JAK-STAT signaling, other virally-stimulated genes are upregulated through the IRF3 and NF-κB pathways [1]. These genes can be called virus stimulated genes (VSGs), and IFNs themselves are VSGs. Interestingly, VSGs have natural anticancer activities, and they may cause diseases in humans when induced chronically. To understand their role in host defense and pathogenesis, excellent model systems have been developed [2]. However, there are still important gaps in our understanding of VSGs and host defense. Recently, RNA sequencing techniques have allowed the discovery of novel VSGs that also include non-coding RNAs [3]. This Special Issue of *Viruses* will explore the impact of VSGs on the outcome of virus infection and the role of these genes within the infected cells and organism. We have focused on the most recent discoveries in VSG research, including the molecular biology of related virus–host interactions. Topics include studies on various steps of gene induction, innate immune responses to virus infection, and the mechanisms of virus immune evasion of related host defense pathways. The clinical presentation of VSG-driven pathology and strategies to use VSGs to cure chronic viral infections are also discussed. In this special Issue, we endeavored to assemble a collection of research papers and reviews that, together, will offer a comprehensive view on new areas of research pertaining to VSGs. 

Majzoub et al. [4] contributed an exciting review, addressing the current knowledge of the innate immune pathways involved in sensing, signaling, and inducing ISGs and VSGs to counter viral infections. Their manuscript specifically discussed Toll-like receptors (TLRs), RIG-I-like receptors (RLRs), and the cGAS-STING pathway, placing their evolution into perspective in diverse organisms and the dynamic co-evolution and diversification of these pathways with respect to viral infections. Liu et al. [5] discussed the mechanisms underlying how herpesviruses can be detected by pattern recognition receptors (PRRs) to induce the expression of interferons (IFNs) and inflammatory cytokines. They also described the ability of herpesviruses to establish persistent infection in the presence of active immune responses involving VSGs. Asmi et al. [6] reviewed Small Ubiquitin-like Modifier (SUMO) protein conjugation as a component of intrinsic and innate immunity, specifically with respect to rhabdoviruses infection and in relation to the production and secretion of interferon. Rhabdoviruses-induced interferon production and the activation of interferon signaling pathways, as well as the expression and functions of viral restriction factors, are discussed. Ashley et al. [7] reported that by using cells engineered to block either the response to, or production of, type-I IFN, the regulation of IFN-independent ISGs was examined in the context of human cytomegalovirus (HCMV) infection. Several ISGs, including IFIT1, IFIT2, IFIT3, Mx1, Mx2, CXCL10, and ISG15 were found to be upregulated transcriptionally following HCMV infection independently of type-I IFN. Furthermore, by using IRF3 knockdown CRISPR/Cas-9 clones, they were able to demonstrate IFN-independent control of ISG expression during HCMV infection of human fibroblasts that is absolutely dependent on IRF3 activation. Finally, He et al. [8] revealed that the THO complex subunit 7 homolog (THOC7) negatively regulates type-I IFN production by promoting TBK1 proteasomal degradation that subsequently decreases IRF3 activation. 

Despite the recent progress in our understanding of these host defense responses, much more is left to be unraveled with respect to the regulation of antiviral responses triggered by distinct viral species. Clearly, IFN is a major component of host defense responses, but there are many ISGs whose antiviral activities remain to be clarified that can be regulated independently of IFN. These new genes would likely be ideal candidates to be targeted through therapeutic intervention as novel antiviral strategies.

We hope this special issue provides a wealth of basic scientific data and introduces new concepts pertaining to ISGs that are regulated independently of IFN signaling.

## References

[1] Wang W., Xu L., Junhong S., Peppelenbosch M.P., Pan Q. (2017). Transcriptional Regulation of Antiviral Interferon-Stimulated Genes. Trends Microbiol..

[2] Ivashkiv L.B., Donlin L.T. (2014). Regulation of type I interferon responses. Nat. Rev. Immunol..

[3] Qiu L.P., Wang T., Tang Q., Li G., Wu P., Chen K. (2018). Long Non-coding RNAs: Regulators of Viral Infection and the Interferon Antiviral Response. Front. Microbiol..

[4] Majzoub K., Wrensch F., Baumert T.F. (2019). The Innate Antiviral Response in Animals: An Evolutionary Perspective from Flagellates to Humans. Viruses.

[5] Liu Q., Rao Y., Tian M., Zhang S., Feng P. (2019). Modulation of Innate Immune Signaling Pathways by Herpesviruses. Viruses.

[6] El Asmi F., Brantis-de-Carvalho C.E., Blondel D., Chelbi-Alix M.K. (2018). Rhabdoviruses, Antiviral Defense, and SUMO Pathway. Viruses.

[7] Ashley C.L., Abendroth A., McSharry B.P., Slobedman B. (2019). Interferon-Independent Upregulation of Interferon-Stimulated Genes during Human Cytomegalovirus Infection is Dependent on IRF3 Expression. Viruses.

[8] He T.-S., Xie T., Li J., Yang Y.-X., Li C., Wang W., Cao L., Rao H., Ju C., Xu L.-G. (2019). THO Complex Subunit 7 Homolog Negatively Regulates Cellular Antiviral Response against RNA Viruses by Targeting TBK1. Viruses.

